# Development and verification of a nomogram for recurrence risk of Benign Paroxysmal Positional Vertigo in middle-aged and older populations

**DOI:** 10.3389/fneur.2024.1483233

**Published:** 2024-12-13

**Authors:** Bo Tang, Chuang Zhang, Dan Wang, Minghua Luo, Yuqin He, Yao Xiong, Xiaojun Yu

**Affiliations:** Department of Neurology, First Hospital of Changsha, Changsha, China

**Keywords:** risk factors, Benign Paroxysmal Positional Vertigo, nomogram, recurrence, BPPV

## Abstract

**Background:**

Benign Paroxysmal Positional Vertigo (BPPV) is the most common cause of peripheral vertigo, with frequent recurrence, particularly pronounced among middle-aged and elderly populations, significantly affecting patients’ quality of life. This study aimed to identify predictive factors for recurrence in middle-aged and older patients with BPPV and to develop a nomogram prediction model based on these predictors.

**Methods:**

This retrospective study included 582 participants aged ≥45 years who were selected from the electronic medical records system of the First Hospital of Changsha between March 2021 and March 2024. Randomly chosen participants (*n* = 407, 70%) constituted the training group, whereas the remaining participants (*n* = 175, 30%) formed the validation group. This study used LASSO binomial regression to select the most predictive variables. A predictor-based nomogram was developed to calculate the risk of BPPV recurrence. The performance of the nomogram was evaluated using the area under the receiver operating characteristic curve (AUC) and calibration curves with 1,000 bootstrap resampling validations. Decision curve analysis (DCA) was conducted to assess the clinical usefulness of the nomogram.

**Results:**

According to findings from least absolute shrinkage and selection operator (LASSO) binomial regression and logistic regression screening, older age, higher levels of uric acid (UA) and homocysteine (HCY), diabetes, migraine, anxiety, and insomnia were identified as independent factors associated with an increased recurrence risk of BPPV. A nomogram model for predicting recurrence risk was developed based on these predictors. The nomogram achieved an AUC (C-statistic) of 0.8974 (95% CI: 0.8603–0.9345) in the training group and 0.8829 (95% CI: 0.8253–0.9406) in the validation group. Calibration curves, after 1,000 bootstrap resamples, demonstrated good agreement between the predicted and actual probabilities in the development and validation cohorts. DCA indicated that the nomogram had clinical utility.

**Conclusion:**

The nomogram model incorporating age, UA, HCY, diabetes, migraine, anxiety status, and insomnia demonstrated a strong predictive capability for estimating the probability of BPPV recurrence in middle-aged and elderly patients. This tool is valuable for identifying individuals at high risk of BPPV recurrence and can aid physicians in making informed treatment decisions aimed at reducing recurrence rates.

## Introduction

Benign Paroxysmal Positional Vertigo (BPPV) is an idiopathic vestibular disorder that results from improper activation of the semicircular canal ampullae by free-floating otoconia originating from the utricle (1). This condition is characterized by brief episodic vertigo and nystagmus triggered by changes in head position (2). As one of the most common vestibular disorders, BPPV is the most common peripheral vestibular condition encountered in neurotology clinics, accounting for approximately 20–30% of all vestibular complaints (3, 4), often occurring in middle-aged and elderly patients (2). Following the canalith repositioning maneuver (CRM), which is the preferred treatment for BPPV, over 95% of cases can be successfully resolved (5). However, patients with BPPV frequently experience relapse even after successful treatment, with recurrence rates ranging from 10 to 30% (6, 7), and the likelihood of recurrence over 10 years can be as high as 50% (8). Patients with BPPV are more prone to future ischemic strokes, dementia, and fractures, which significantly diminish their quality of life, particularly among older people (9–11). These complications also increase the burden on patients and the healthcare system.

However, the underlying cause of BPPV recurrence remains unclear. In recent decades, numerous studies have explored the risk factors for BPPV recurrence, including female sex, osteoporosis, vascular risk factors, head trauma, and other potential contributors (3, 7, 12–15). Nevertheless, the identified risk factors varied between studies, and the impact of each risk factor on recurrence differed. Therefore, developing a predictive model that incorporates as many potential risk factors as possible is crucial for assessing the risk of BPPV recurrence in middle-aged and older populations. This approach aids in the early and accurate identification of recurrence risk, allowing for the timely implementation of preventive strategies.

Currently, nomograms serve as tools that integrate various predictors into graphical instruments for statistical models, offering the predicted probability of a clinical event or specific endpoint outcome (16, 17). Therefore, the development of a nomogram can help predict the probability of BPPV recurrence and provide timely, individualized, and comprehensive prevention recommendations. However, effective and sensitive predictive models for BPPV recurrence in middle-aged and elderly individuals are currently lacking.

Therefore, this study aimed to analyze the risk factors of BPPV recurrence in middle-aged and elderly individuals following successful treatment. Additionally, this study aimed to construct a nomogram prediction model to provide patients with optimal and timely clinical decision-making and preventive recommendations.

## Methods

### Study design and source of data

This retrospective cohort study included 582 patients consecutively diagnosed with BPPV and admitted to the Neurology Department of the First Hospital of Changsha between March 2021 and March 2024. In our study, the licensed neurologist utilized the following approaches to ensure that BPPV was accurately diagnosed: (1) Clinical Criteria: Diagnosis was based on established clinical criteria outlined in the guidelines from the Bárány Society (18). We confirmed the presence of characteristic symptoms, including episodic vertigo triggered by specific head movements, typically lasting less than 1 minute. (2) Clinical Examination: All participants underwent a thorough clinical examination. We specifically performed the Dix-Hallpike maneuver and/or the supine roll test to identify the presence of nystagmus consistent with BPPV. The direction and characteristics of the nystagmus were carefully analyzed. (3) Exclusion of Other Conditions: We excluded other potential causes of vertigo by conducting a comprehensive medical history and physical examination, along with additional tests (e.g., audiometry, MRI) when necessary. And the exclusion criteria were as follows: (1) patients with sudden deafness or other vestibular diseases; (2) individuals with hearing impairment; (3) those who experienced head trauma within the last month; (4) patients with systemic musculoskeletal diseases; (5) patients with severe organic diseases; (6) those with cervical vertigo, brain space-occupying lesions, cerebrovascular malformations, or central vertigo; and (7) individuals with involvement of multiple semicircular canals or superior semicircular canal otolithiasis.

Using the “GetTable_comparet” method in R software, we randomly split the overall dataset into training and validation cohorts in a 7:3 ratio, resulting in 407 patients in the training cohort and 175 patients in the validation cohort. The Ethics Committee of the First Hospital of Changsha approved this study. The study was carried out in accordance with the guidelines of Transparent Reporting of a multivariable prediction model for Individual Prognosis or Diagnosis (TRIPOD) (19). The requirement for informed consent was waived in accordance with national regulations and agency guidelines Owing to the retrospective nature of our study. Patients’ identifying information (e.g., names, addresses, and phone numbers) was removed from the dataset throughout the study to ensure participants’ anonymity and to protect privacy. The data were securely stored using encryption and access controls.

### Candidate predictor variables

Based on preliminary observations from clinical practice, references from previous literature (20), and the availability of retrospective study data, we selected potential risk predictors that may influence the recurrence of BPPV. We collected electronic medical record data during the patients’ hospital stays, including demographics, lifestyle factors, comorbidities, clinical symptoms, vestibular function test results, and laboratory findings. Demographic variables included sex, BMI, and age at BPPV onset. Lifestyle factors included smoking habits and alcohol consumption. The medical histories of the participants revealed comorbidities such as hypertension, diabetes, migraine, Meniere’s disease (MD), anxiety, insomnia, and osteoporosis. The laboratory examination variables included hyperlipidemia, thyroid dysfunction, globulin levels, serum calcium ion levels, uric acid (UA), and homocysteine (HCY). The duration of illness in the patients was calculated in days, and we categorized the duration into two groups using a cutoff of 3 days. Additionally, we collected data regarding the season of illness (spring, summer, autumn, and winter) for classification purposes.

We recorded the results of vestibular function tests, including the *C*-test, cupulolithiasis, and common types of BPPV. The *C*-test was conducted using VNG (videonystagmography) with the following sequence (21): irrigation of the right ear with air at 24°C, irrigation of the left ear with air at 24°C, irrigation of the right ear with air at 50°C, and irrigation of the left ear with air at 50°C. A positive *C*-test result was considered when the semicircular canal paresis (CP) value exceeded 25%.

This study aimed to predict whether patients will experience recurrence after undergoing CRM. We have dedicated staff who follow up with patients diagnosed with BPPV who have received CRM, using methods such as telephone follow-ups, outpatient visits, and readmission assessments. Patients who were readmitted or return for outpatient care due to BPPV symptoms more than 2 weeks after successful repositioning, as well as those identified as having BPPV after being treated for dizziness at other hospitals after 2 weeks based on our telephone follow-ups, were uniformly classified as having recurrent BPPV (6). In this study, we excluded patients who had a history of head trauma or whiplash injuries, those with incomplete clinical histories, and individuals diagnosed with “persistent” Benign Paroxysmal Positional Vertigo (BPPV). “Persistent” BPPV is defined as the absence of symptom remission or nystagmus after 2 weeks or following five repositioning maneuvers (6).

### Statistical analysis

Statistical methods were used to evaluate differences between the training and validation cohorts, including (1) normality tests, such as skewness and kurtosis tests for continuous variables; (2) the Mann–Whitney *U*-test for continuous variables that did not exhibit a normal distribution, with median and interquartile range (IQR) values reported; and (3) the chi-square test for categorical variables, presented as percentages.

Subsequent analyses were conducted using the R software (version 4.4.0) with various packages, including rms, car, glmnet, pROC, regplot, and rmda. First, to avoid collinearity among the included covariates and to identify the optimal predictive risk factors, we screened the potential risk factors for recurrent BPPV using LASSO regression. LASSO binomial regression is a statistical technique that helps identify key predictors for a binary outcome by selectively including variables while minimizing complexity. It adds a penalty to the regression to prevent overfitting, making the model more reliable and easier to interpret. This method helps avoid collinearity among covariates and selects optimal predictive risk factors (22). Variables selected by LASSO regression were included in multivariate logistic regression analysis to develop a nomogram prediction model. Receiver-operating characteristic (ROC) curves were used to assess the sensitivity and specificity of the nomogram (23). Calibration curve and decision curve analyses (DCA) were used to evaluate the predictive performance of the nomogram. Decision curve analysis (DCA) was conducted to assess the clinical utility of the nomogram and calculate the net benefits across various threshold probabilities (24). We employed the 1,000-bootstrap resampling validation method for internal validation to enhance the accuracy and reliability of the model. For validation, the ROC curve, calibration curve, and decision curve analyses (DCA) were performed using the same methods as described previously. Statistical significance was set at *p* < 0.05, indicating statistical significance.

## Results

**Figure 1:**
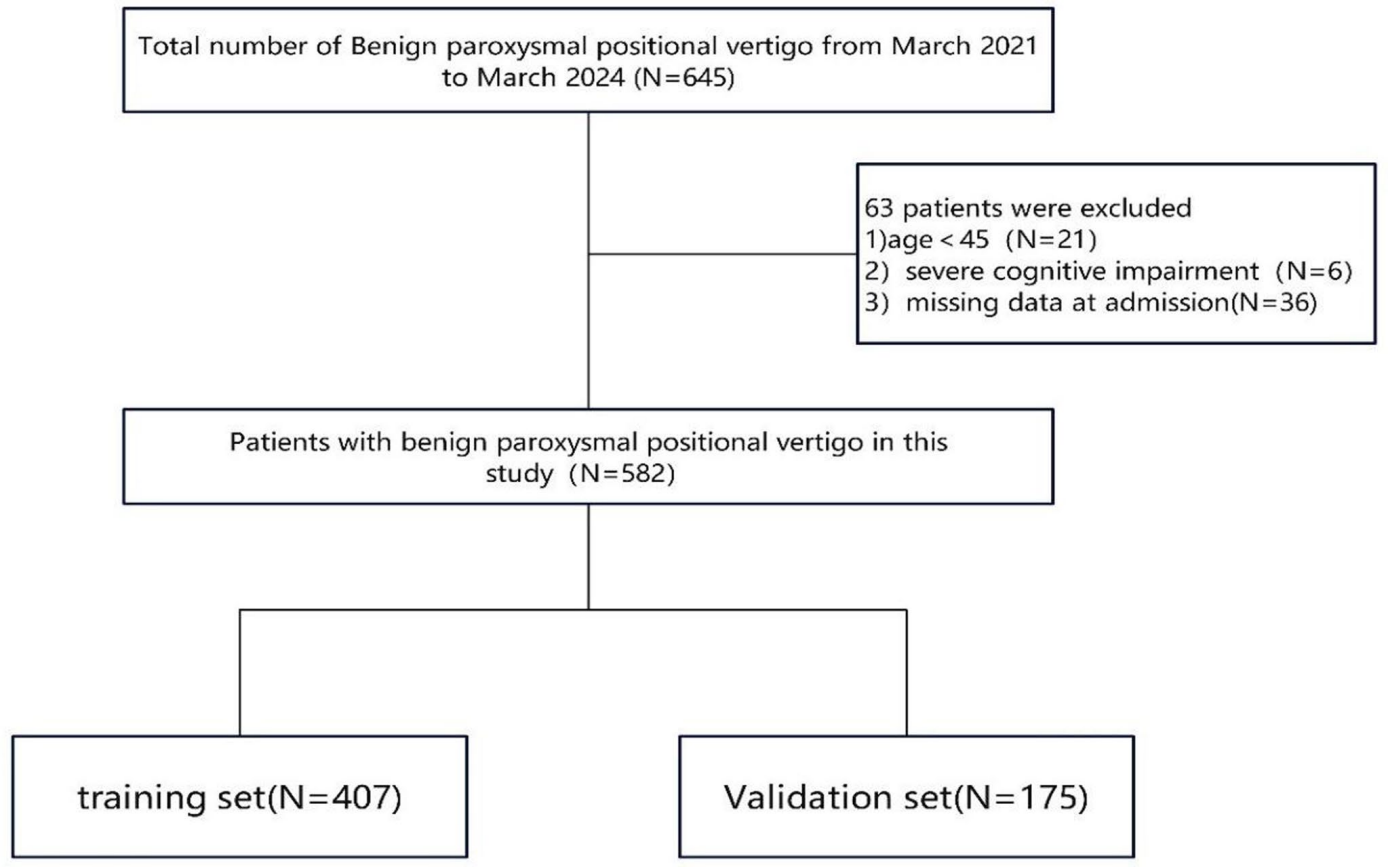
The flow chart of patient recruitment concisely outlines the sequential application of inclusion and exclusion criteria, ultimately defining the final study cohort.

A total of 645 patients diagnosed with BPPV met the initial inclusion criteria. After reviewing medical records and conducting telephone interviews, we excluded 21 patients aged <45 years, six patients with severe cognitive impairment who could not cooperate in conversations, and 36 patients who were lost to follow-up or had missing data. Finally, 582 eligible participants were included in the analysis, with 407 and 175 participants in the training and validation cohorts, respectively (Figure 1).

### Basic characteristics

Table 1 summarizes the baseline characteristics of the training and validation datasets used in this study. The training set comprised 407 middle-aged and elderly patients, whereas the validation set included 175 similar patients. Importantly, there were no statistically significant differences between the training and validation groups for all characteristics (*p* > 0.05), indicating that the grouping of all BPPV patients was reasonable.

**Table 1:** Characteristics of patients in the training and validation sets.

Variables	Training set (*n* = 407)	Validation set (*n* = 175)	*p*-value
Gender (*n*%)			
Male	119 (29.24%)	58 (33.14%)	
Female	288 (70.76%)	117 (66.86%)	0.4
Age (years)	66.85 ± 9.43	66.56 ± 8.81	0.74
BMI (kg/m^2^)	21.63 ± 2.55	21.52 ± 2.7	0.51
Smoke (*n*%)			
Yes	25 (6.14%)	9 (5.14%)	
No	382 (93.86%)	166 (94.86%)	0.78
Alcohol drinking (*n*%)			
Yes	35 (8.6%)	9 (5.14%)	
No	372 (91.4%)	166 (94.86%)	0.2
Season (*n*%)			
Spring	124 (30.47%)	63 (36%)	
Summer	100 (24.57%)	47 (26.86%)	
Autumn	72 (17.69%)	30 (17.14%)	
Winter	111 (27.27%)	35 (20%)	0.26
Course of disease (*n*%)			
≤3 days	254 (62.41%)	114 (65.14%)	
>3 days	153 (37.59%)	61 (34.86%)	0.59
Diabetes (*n*%)			
Yes	64 (15.72%)	30 (17.14%)	
No	343 (84.28%)	145 (82.86%)	0.76
Hypertension (*n*%)			
Yes	188 (46.19%)	89 (50.86%)	
No	219 (53.81%)	86 (49.14%)	0.35
Migraine (*n*%)			
Yes	41 (10.07%)	15 (8.57%)	
No	366 (89.93%)	160 (91.43%)	0.68
Hyperlipemia (*n*%)			
Yes	124 (30.47%)	52 (29.71%)	
No	283 (69.53%)	123 (70.29%)	0.93
MD (*n*%)			
Yes	50 (12.29%)	21 (12%)	
No	357 (87.71%)	154 (88%)	1.0
Thyroid dysfunction (*n*%)			
Yes	47 (11.55%)	24 (13.71%)	
No	360 (88.45%)	151 (86.29%)	0.55
Anxiety (*n*%)			
Yes	68 (16.71%)	33 (18.86%)	
No	339 (83.29%)	142 (81.14%)	0.61
Insomnia (*n*%)			
Yes	105 (25.8%)	50 (28.57%)	
No	302 (74.2%)	125 (71.43%)	0.55
Osteoporosis (*n*%)			
Yes	99 (24.32%)	43 (24.57%)	
No	308 (75.68%)	132 (75.43%)	1.0
UA (μmol/L)	343.05 ± 47.46	341.96 ± 48.36	0.8
Globulin (g/L)	23.22 ± 3.07	22.96 ± 2.89	0.33
Calcium ion (mmol/L)	2.5 ± 0.24	2.45 ± 0.26	0.06
*C*-test			
Positive	27 (6.63%)	16 (9.14%)	
Negative	380 (93.37%)	159 (90.86%)	0.37
Cupulolithiasis (*n*%)			
Yes	32 (7.86%)	12 (6.86%)	
No	375 (92.14%)	163 (93.14%)	0.8
Semicircular canal involvement (*n*%)			
Left horizontal semicircular canal	64 (15.72%)	34 (19.43%)	
Right horizontal semicircular canal	72 (17.69%)	25 (14.29%)	
Left posterior semicircular canal	166 (40.79%)	68 (38.86%)	
Right posterior semicircular canal	105 (25.8%)	48 (27.43%)	0.55
HCY (μmol/L)	15.91 ± 7.04	15.27 ± 6.86	0.19
Recurrence (*n*%)			
Yes	114 (28.01%)	54 (30.86%)	
No	293 (71.99%)	121 (69.14%)	0.55

### Selection of variables and construction of the nomogram

**Figure 2:**
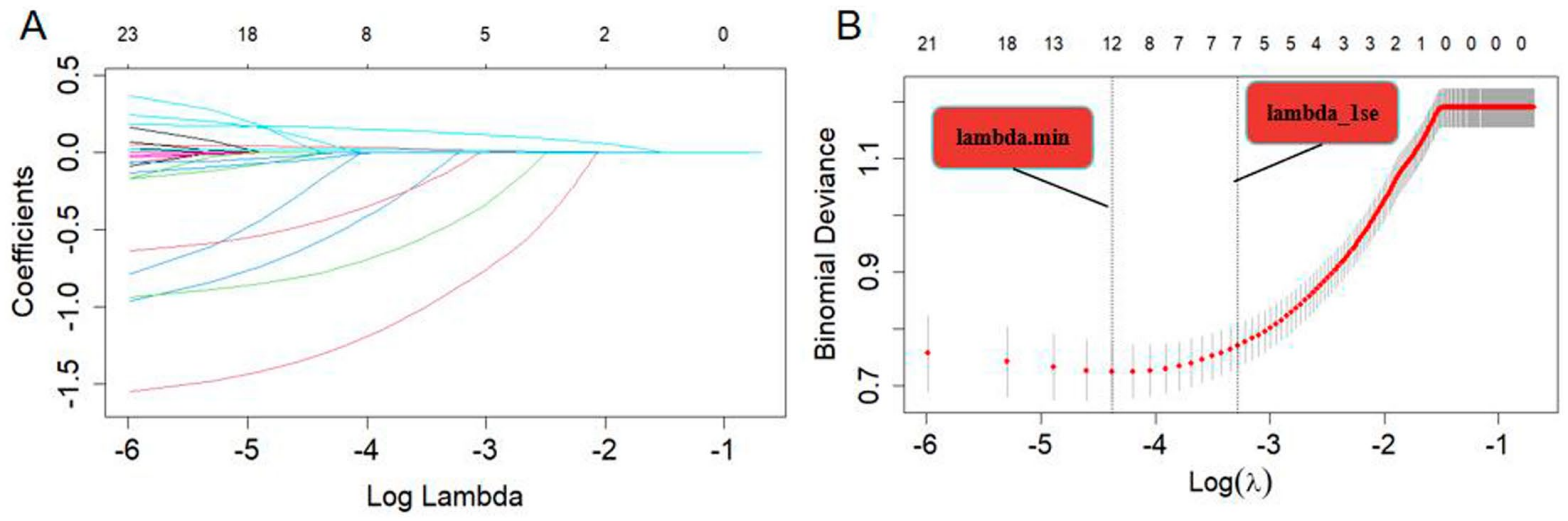
**(A)** LASSO coefficient profiles of the 23 risk factors. **(B)** Risk factors were selected using LASSO regression analysis. The two dotted lines indicate the optimal scores according to the minimum criteria (including age, smoking, Alcohol drinking, diabetes, hypertension, migraine, hyperlipidemia, anxiety, insomnia, osteoporosis, UA, and HCY at minimum criteria; age, diabetes, migraine, anxiety, insomnia, UA, and HCY at 1-se criteria).

**Figure 3:**
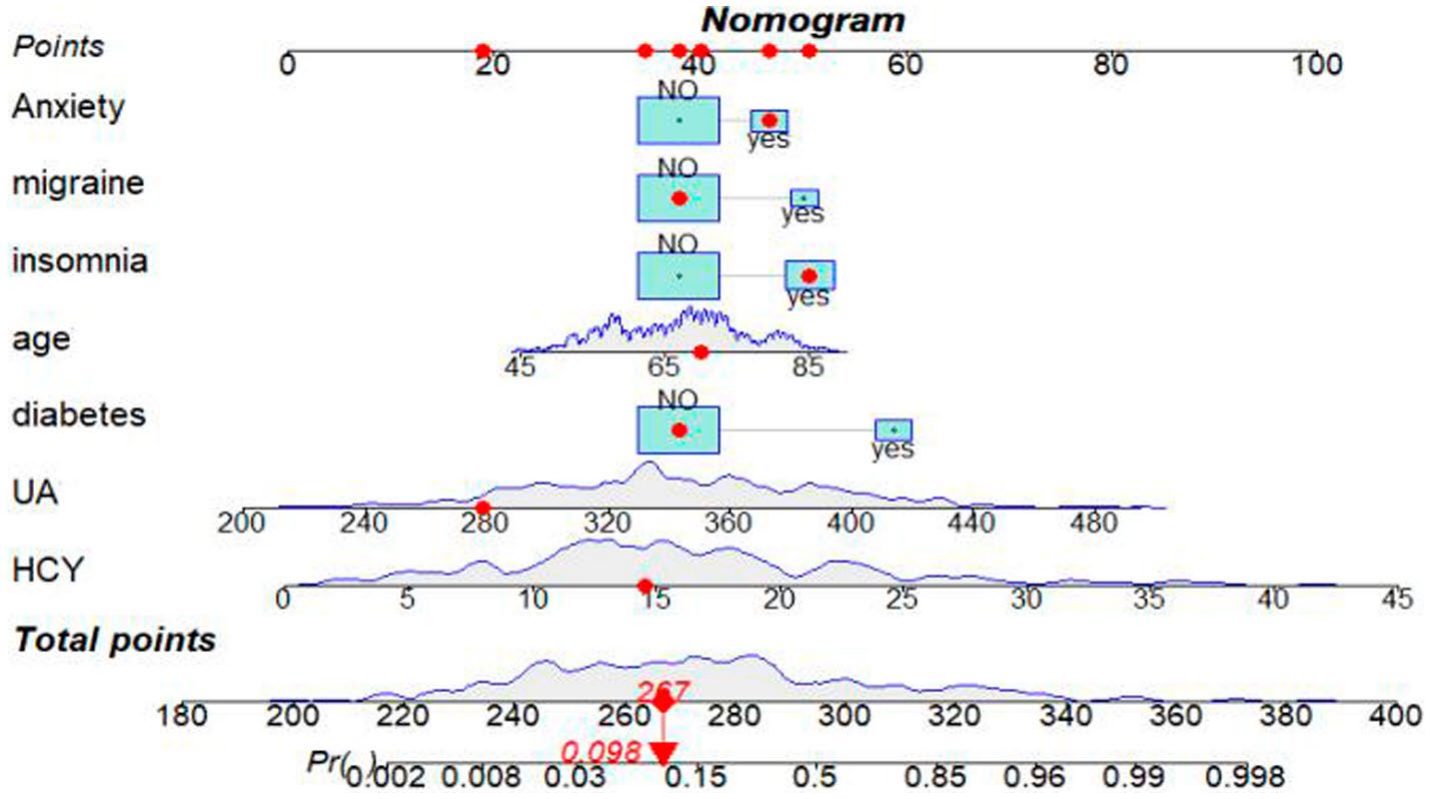
Nomogram for predicting the probability of BPPV recurrence after CRM. A red dot on the nomogram represents the specific characteristics of a patient.In this example, a 70-year-old individual with a history of insomnia and anxiety, but no migraines or diabetes, has a UA level of 279 μmol/L and a HCY level of 14.57 μmol/L. The calculated sum of these specific points is 267, which corresponds to a position on the total point line. From this point, a solid line is drawn vertically down to the survival axis, indicating a recurrence probability of BPPV of 9.8% for this patient.

Variable selection was performed using LASSO regression, as illustrated in Figure 2. Seven variables were significantly associated with BPPV recurrence (*p* < 0.05). These variables include age (years), diabetes (Yes), migraine (Yes), anxiety (Yes), insomnia (Yes), UA (μmol/L), and HCY (μmol/L). A nomogram was constructed using these significant factors, as shown in Figure 3. Detailed information on the relationship between these variables and BPPV recurrence is provided in Table 2.

**Table 2:** Identification of independent risk factors for the recurrence of Benign Paroxysmal Positional Vertigo in middle-aged and older populations using multivariable logistic regression: findings from the training dataset.

Variables	OR	95% CI	*p*-value
Age	1.052	1.012–1.096	0.012
Diabetes	5.261	2.393–11.896	<0.001
Anxiety	2.099	0.882–5.004	0.039
Migraine	2.862	1.038–7.859	0.041
Insomnia	2.693	1.408–5.198	0.003
UA	1.024	1.016–1.033	<0.001
HCY	1.218	1.153–1.282	<0.001

### Evaluation and validation of the nomogram

**Figure 4:**
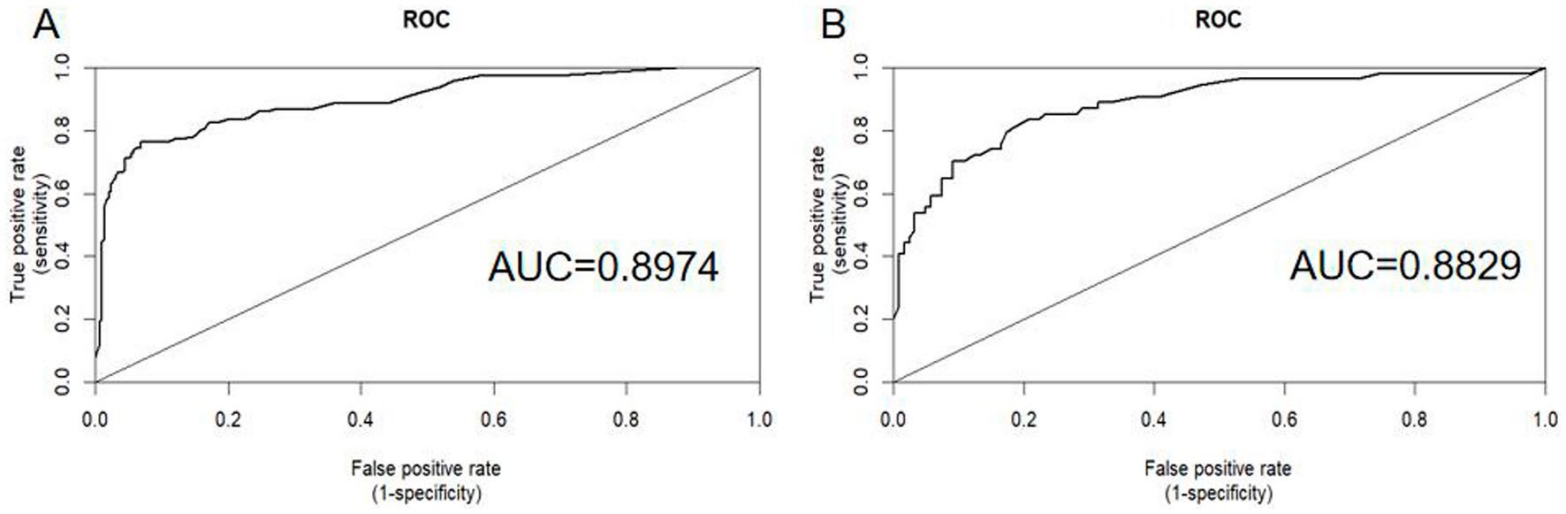
ROC curves were generated to assess the predictive performance of the nomogram for the recurrence probability of BPPV following CRM. Panel A displays the ROC curve for the training set, whereas Panel B shows the ROC curve for the validation set. AUC, area under the ROC curve; ROC, receiver operating characteristic.

The nomogram was validated using AUC-ROC. In the training set (Figure 4A), the AUC-ROC was 0.8974 (95% CI: 0.8603–0.9345), whereas in the validation set (Figure 4B), it was 0.8829 (95% CI: 0.8253–0.9406).

**Figure 5:**
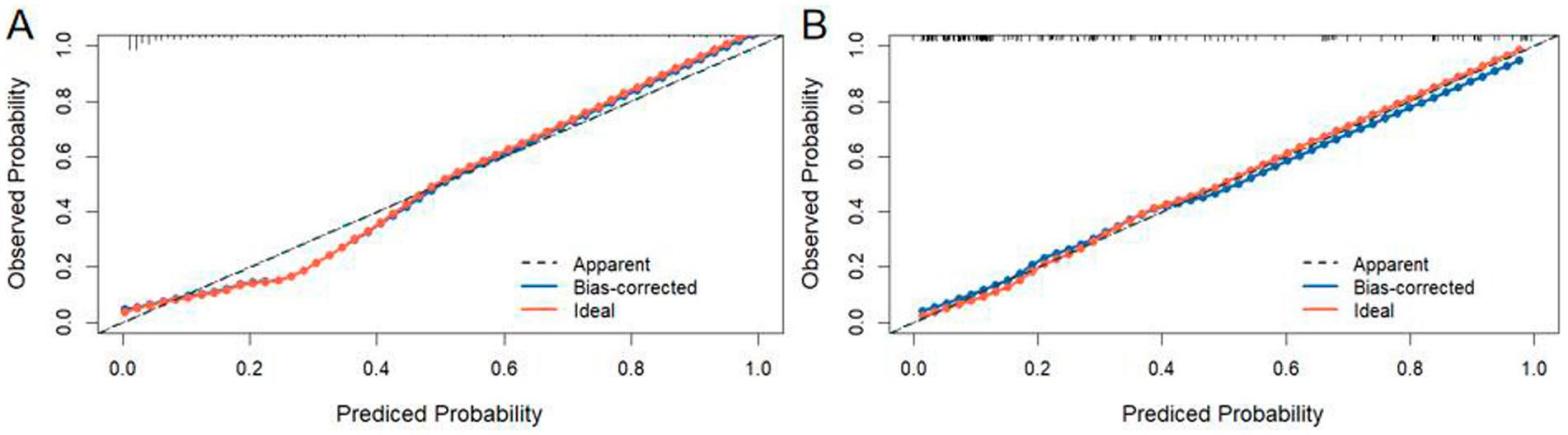
Calibration curves of the nomogram were plotted separately for the training set **(A)** and the validation set **(B)**.

The nomogram model was calibrated using calibration plots. These plots demonstrated excellent agreement between the predicted and observed outcomes for the training (Figure 5A) and validation sets (Figure 5B).

**Figure 6:**
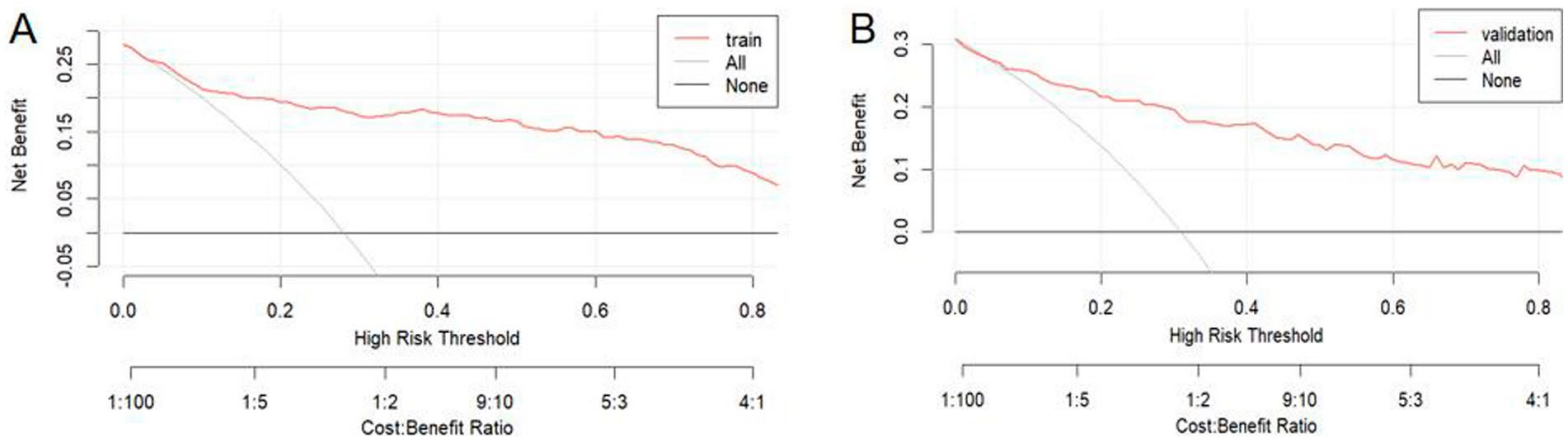
Decision curve analysis was performed for the training set **(A)** and the validation set **(B)**. In these plots, a horizontal line signifies that no patients are predicted positive, resulting in a net benefit of zero. Conversely, an oblique line indicates that all patients are predicted positive. A backslash with a negative slope represents the net benefit.

DCA was used to evaluate the clinical utility of the nomogram (Figures 6A,B). The results confirmed the robust clinical applicability of the nomogram for predicting the probability of BPPV recurrence, as evidenced by the wide and practical range of threshold probabilities across the training and validation sets.

## Discussion

Currently, no predictive model has been identified for BPPV recurrence. Therefore, developing a model for predicting BPPV recurrence is essential. This study is the first to create a nomogram for predicting the risk of BPPV recurrence. In this study, we developed a straightforward, valid, and clinically useful model for predicting the probability of BPPV recurrence in middle-aged and older patients. The AUC for the training and validation groups were 0.8974 and 0.8829, respectively, indicating that the nomogram-predictive model demonstrated good accuracy and stability. This nomogram model incorporates easily obtainable risk factors, including age, diabetes, migraine, anxiety, insomnia, UA, and HCY. All of these variables can be readily collected during the hospital stay of patients with BPPV. Using this model, clinicians can quickly calculate recurrence risk in middle-aged and elderly patients with BPPV. Patients at high risk of recurrence may benefit from more preventive interventions and early treatment.

Nomograms have emerged as advanced and straightforward prediction tools. They offer a visual representation of a statistical predictive model that generates the numerical probability of a clinical event, making them more accurate than conventional methods that use odds ratios (ORs) (25). This nomogram combines seven variables and assigns appropriate weights to each variable based on its prognostic value. It provides a personalized and highly accurate estimation of the risk of BPPV recurrence, making it easy to use. Thus, physicians can assess a patient’s high risk of BPPV recurrence, recommend lifestyle and behavioral changes, actively manage risk factors, and implement appropriate treatment measures to reduce the likelihood of recurrence.

Our recurrence rate of 28.9% was consistent with that reported in the literature, which ranges from 7 to 50% (3, 6, 7). Previous studies have identified several risk factors for BPPV recurrence. Two recent population-based retrospective cohort studies have indicated that patients with anxiety have a higher risk of developing BPPV compared to those without anxiety (26, 27). The higher risk of recurrence could be owing to the notably reduced efficacy of initial CRM treatment in patients with BPPV and anxiety, resulting in a higher risk of recurrence several months after treatment (28). Recovery from balance disorders, including BPPV, involves habituation and relearning. This process involves various structures, mechanisms, and activities, such as neurophysiological adaptation, desensitization to dizziness sensations, and restoration of automatic perception and control of orientation, all of which are influenced by psychological factors (29). Therefore, anxiety can affect the recovery process from BPPV, leading to prolonged vertigo or dizziness (29).

Furthermore, BPPV symptoms are often unpredictable and uncontrollable, leading to uncertainty and fear of experiencing new symptoms (30). Yardley and colleagues (31) outlined three distinct clusters of concerns among patients with vertigo: fear of losing control, worry about serious illnesses, and anticipation of severe vertigo attacks. Hence, we hypothesized that psychological conditions could complicate and disrupt habituation and coping processes, potentially heightening vertigo symptoms through amplified autonomic responses (29).

Our study found that insomnia (OR = 2.693; *p* = 0.003) was associated with an increased risk of BPPV recurrence, which is consistent with the findings of Wang Y (32). Previous studies have also suggested that insomnia could lead to BPPV by triggering neuroendocrine dysfunction, marked by elevated cortisol levels and increased sympathetic nervous system activity (33–35), along with inflammatory activation of vestibular neurons (33, 36).

Older age is a well-recognized risk factor for BPPV recurrence and has been consistently noted in most studies of BPPV. As age progresses, demineralization occurs, leading to the breakdown, fragmentation, and detachment of utricular otoconia, which in turn causes BPPV (37). Another possible reason is that vestibular function deteriorates with age, leading to an abnormal dynamic balance between the production and absorption of otoliths. Additionally, after repositioning, otolith fragments may remain in the ear, further delaying the adaptation of the central system (38). Furthermore, the increased recurrence rate in elderly patients may be attributed to decreased daily activity levels, restricted mobility, fatigue, and a higher incidence of falls (39–41). However, whether age is an independent prognostic factor remains controversial. Some studies (42, 43) have found that BPPV recurrence is not associated with age, whereas others (15, 39) indicate that the recurrence rate of BPPV increases with advancing age. Large-scale studies have demonstrated that patients aged over 40 (44) or 50 years (40), particularly those in their sixth (7) or seventh decade of life (4), are more likely to experience recurrence. Piccioti et al. (15) demonstrated that individuals older than 65 years have a 1.6 times higher risk of BPPV recurrence than those younger than 65 years. They also highlighted that the presence of comorbidities, such as hypertension, diabetes, and vascular diseases, might contribute to increased recurrence rates among elderly patients.

Migraine is strongly associated with various forms of vertigo, including BPPV. A recent survey indicated that BPPV is twice as common in individuals with migraines (45). Additionally, a recent meta-analysis of the existing literature identified migraine as a factor that predisposes individuals to BPPV recurrences (46). Our study also found that migraine (OR, 2.862; *p* = 0.041) was associated with an increased risk of BPPV recurrence. The pathophysiological link between migraines and BPPV is not fully understood. Previous studies have confirmed that repeated vasospasms can affect the microvasculature of the inner ear, leading to vascular damage and, consequently, the recurrence of BPPV (47, 48). Furthermore, vasospasm-induced suppression of the inner ear microvasculature may result in cochlear symptoms, such as hearing disturbances and vestibular symptoms (49).

This study found that diabetes played a significant role among the factors contributing to BPPV recurrence (OR = 5.261; *p* < 0.001). This conclusion is consistent with those of many previous studies (50–52). Chronic hyperglycemia can lead to microvascular damage and histopathological changes in peripheral neuropathy, which negatively impacts the blood supply to the inner ear via terminal branches, thereby affecting the vestibular function of patients. Additionally, glucose can enter the endolymph, altering the pH and changing the solubility of otoconia, leading to abnormal metabolism and detachment of utricular otoconia, which in turn results in disease recurrence (4, 53). However, other studies have found no association between diabetes and the recurrence of BPPV (28, 41, 54). Therefore, further studies are required to determine the effects of diabetes on BPPV recurrence.

This study also identified UA as an independent risk factor for BPPV recurrence, which is consistent with the conclusions of a previous meta-analysis (55). UA has unique oxidative and antioxidative properties and possibly acts as a novel inflammatory factor involved in oxidative stress, inflammation, and metabolic processes. Elevated serum uric acid levels may trigger inflammation in the otoconial gelatinous matrix, release inflammatory mediators, induce the production of reactive oxygen species (ROS), and damage the vascular system. These processes, in turn, impair endothelial function and blood supply to the inner ear, leading to BPPV recurrence (55, 56). In addition, research indicates that higher levels of uric acid entering the lymphatic fluid can lower the pH of the endolymph, thereby preventing the dissolution of otoconia fragments, which in turn may trigger BPPV (57). However, this study also indicated that HCY could increase the risk of BPPV recurrence. As an intermediate product of methionine metabolism, HCY primarily damages the vascular endothelium and promotes the participation of vascular smooth muscle cells in coagulation, leading to microcirculatory disorders of the inner ear. High HCY levels can induce atherosclerosis and stenosis in the arteries supplying blood to the inner ear (58, 59), resulting in inner ear ischemia, thereby increasing the risk of BPPV.

It is noteworthy that previous studies (20, 38) have indicated that hypertension, hyperlipidemia and stroke are risk factors for BPPV, while this study confirms that diabetes, hyperuricemia, and hyperhomocysteinemia are also risk factors for the recurrence of BPPV. The mechanisms by which these factors lead to BPPV onset are similar to those of hyperlipidemia, hypertension, and stroke, as they cause narrowing and spasm of cerebral arteries, resulting in vestibular dysfunction that is sensitive to ischemia, ultimately leading to abnormal otolith metabolism and the detachment of otoliths. The differences in study results may be attributed to interactions among sample characteristics, regional characteristics, potential risk factors, and the accessibility of medical resources. Therefore, future research needs to expand the sample size and conduct prospective studies to validate these findings.

Certainly, this study has several limitations that need to be taken into account. First, our data were gathered retrospectively from a single center potentially restricting the statistical robustness of the findings. Additionally, owing to the retrospective design of the study, some admission data were not available. For example, previous study (60) indicated that low vitamin D levels may increase the risk of BPPV recurrence, potentially due to its effect on calcium metabolism, which can impair the synthesis and function of otoconia composed of calcium carbonate, leading to their dislodgement and reformation of BPPV. However, as this study is retrospective, more than half of the patients did not undergo vitamin D level assessment, resulting in significant missing values. Therefore, we chose not to include this variable in the analysis, which constitutes a limitation of this study. This limitation highlights the need for future research to incorporate vitamin D assessment in prospective studies to better understand its role in BPPV recurrence. Second, our model has not been tested on external cohorts. It is essential to evaluate the utility of our new nomogram in future prospective studies and validate it through multicenter investigations. Third, we should consider the probability that a bias in our work may have resulted from the fact that we excluded participants who we were unable to contact by phone, possibly without recurrence, leading to an overestimation of the value. The accuracy of some of our results may be limited by significant heterogeneity or the limited number of included studies. Therefore, further research is needed to confirm these results. Finally, our nomogram was developed using only seven available predictors. Further investigations are required to determine whether expanding the number of variables enhances the nomogram. However, increasing the complexity of a nomogram can reduce its clinical utility. Despite these limitations, we successfully identified seven prognostic factors for BPPV recurrence in the middle-aged and older populations. In future, the findings of this study could serve as a reference for predicting the risk of BPPV recurrence across all age groups.

## Conclusion

This study developed a nomogram incorporating demographic traits, vascular risk factors, emotional aspects, and lifestyle behaviors to predict the risk of BPPV recurrence in middle-aged and older adults. Validation demonstrated the accurate and stable predictive performance of the nomogram. This tool aids physicians in making informed treatment decisions to minimize BPPV recurrence.

## Data Availability

The raw data supporting the conclusions of this article will be made available by the authors, without undue reservation.
